# Short‐latency afferent inhibition is reduced with cold‐water immersion of a limb and remains reduced after removal from the cold stimulus

**DOI:** 10.1113/EP091896

**Published:** 2024-08-27

**Authors:** Eden T. Delahunty, Leanne M. Bisset, Justin J. Kavanagh

**Affiliations:** ^1^ Menzies Health Institute Queensland Griffith University Queensland Australia

**Keywords:** afferent inhibition, cold water immersion, transcranial magnetic stimulation

## Abstract

**Abstract:**

The experience of pain that is induced by extremely cold temperatures can exert a modulatory effect on motor cortex circuitry. Although it is known that immersion of a single limb in very cold water can increase corticomotor excitability it is unknown how afferent input to the cortex shapes excitatory and inhibitory processes. Therefore, the purpose of this study was to examine motor‐evoked potentials (MEP), short‐latency afferent inhibition (SAI) and long‐latency afferent inhibition (LAI) in response to immersion of a single hand in cold water. Transcranial magnetic stimulation (TMS) was used to assess MEPs, and peripheral nerve stimulation of the median nerve paired with TMS was used to measure SAI and LAI in motor circuits of the ipsilateral hemisphere. Measurements were obtained from electromyography (EMG) of the first dorsal interosseous (FDI) at baseline, during cold‐water immersion, and during recovery from cold‐water immersion. The intervention caused unconditioned MEPs to increase during exposure to the cold stimulus (*P* = 0.008) which then returned to baseline levels once the hand was removed from the cold water. MEP responses were decoupled from SAI responses, where SAI was reduced during exposure to the cold stimulus (*P* = 0.005) and remained reduced compared to baseline when the hand was removed from the cold water (*P* = 0.002). The intervention had no effect on LAI. The uncoupling of SAI from MEPs during the recovery period suggests that the mechanisms underlying the modulation of corticospinal excitability by sensory input may be distinct from those affecting intracortical inhibitory circuits.

**Highlights:**

- **What is the central question of this study?**
  Does immersion of a limb in very cold water influence corticospinal excitability and the level of afferent inhibition exerted on motor cortical circuits?
- **What is the main finding and its importance?**
  In additional to perception of temperature, immersion in 6°C water also induced perceptions of pain. Motor evoked potential (MEP) amplitude increased during immersion, and short‐latency afferent inhibition (SAI) of the motor cortex was reduced during immersion; however, these responses differed after the limb was removed from the cold stimulus, as MEPs returned to normal levels while SAI remained suppressed.

## 1 INTRODUCTION

The experience of pain, induced by interventions such as thermal stress, exerts a substantial modulatory effect on motor cortex circuitry (Ansari & Tremblay, 2019; Chang et al., 2018; Sato et al., 2013). Cold‐water immersion of a single limb activates A‐delta and C fibres, transmitting nociceptive signals via the spinothalamic tract to the thalamus, which relays them to the primary and secondary somatosensory cortices, anterior cingulate cortex, and insular cortex for processing (Hu et al., 2015; Janig et al., 2009; Lefaucheur, 2019; Shimo et al., 2022). This nociceptive input can engage descending inhibitory pathways, originating from regions such as the periaqueductal grey and rostral ventromedial medulla, to modulate spinal cord transmission and reduce pain perception. Activation of these inhibitory pathways with a short‐term painful stimulus can decrease corticomotor excitability, potentially enhancing motor function by mitigating the disruptive effects of pain on motor control (Burns et al., 2016a; Chowdhury et al., 2022; Rohel et al., 2021; Salo et al., 2019). Similarly, thermal induced pain engages bilateral activation of the primary and secondary somatosensory cortices, with notable activation in the ipsilateral cortex during intense stimuli (Becerra et al., 2009; Craig et al., 2000; Freund et al., 2009). While some noxious thermal stimuli can decrease excitability, there are instances where thermal pain increases motor cortex excitability in the ipsilateral hemisphere, demonstrating a complex and variable response in cortical regions (Chowdhury et al., 2022).

When applied to the motor cortex, transcranial magnetic stimulation (TMS) induces motor evoked potentials (MEP) in muscles, with the amplitude of these MEPs reflecting the net excitability of cortical and spinal motor neurons (Di Lazzaro et al., 2008). The modulation of MEP amplitudes in hand muscles through TMS can be influenced by conditioning electrical stimuli to the contralateral median nerve, a phenomenon that is contingent on the temporal interaction between the afferent stimuli and the TMS pulse (Tokimura et al., 2000; Turco et al., 2018b, 2021). Short‐latency afferent inhibition (SAI), occurring at intervals typically below 30 ms, implicates cholinergic circuits modulating cortical excitability (Celebi et al., 2012; d'Angremont et al., 2024; Hannah, 2020). Conversely, long‐latency afferent inhibition (LAI), characterized by extended intervals between stimuli, is believed to be mediated by different neurotransmitter systems, possibly involving GABAergic pathways (although the exact mechanisms remain unknown) (Turco et al., 2018a; Udupa et al., 2009). Despite the known effects of cold immersion on hemispheric activity, the specific impact on afferent pathways and their consequent influence on the ipsilateral motor cortex output remain unexplored. SAI and LAI are critical mechanisms through which sensory input can modulate motor cortical output, yet their behaviour in the context of cold‐induced pain and temperature changes has not been investigated.

For practical reasons it is difficult to examine cortical activity in the contralateral hemisphere during cold water immersion, as this requires electrical stimulation of a limb in water. Therefore, the purpose of this study was to determine how immersion of a single limb in very cold water (6°C) affects TMS‐evoked MEPs associated with the ipsilateral hemisphere. The strength of afferent inhibition on MEPs from the ipsilateral hemisphere was then determined via SAI and LAI measurements. Our previous work using a similarly cold, single limb intervention identified increased MEPs from both cortical hemispheres (Delahunty et al., 2019), so we hypothesised in the current study that MEP amplitude would increase in the ipsilateral hemisphere during cold water immersion, and then decline to baseline levels during recovery when the limb was removed from the cold water. It was also hypothesised that afferent inhibition assessed via SAI and LAI would be reduced during cold water immersion and then return to baseline levels during recovery when the limb was removed from the cold water.

## 2 METHODS

### 2.1 Participants and ethical approval

Fifteen healthy, recreationally active, participants were recruited into the study (age: 25 ± 2 years, female: 2). Sample size calculations indicated that a sample of 12 healthy individuals were required to achieve a power of 0.80 with an α level of 0.05 and effect size (η^2^) of 0.14 using a repeated measures design (GPower 3.1.9.7). Prior to testing, participants were screened and excluded if they used central nervous system‐acting medications, had a history of neurological dysfunction, or had any recent neuromuscular injury. The dominant hand for each participant was determined using the Oldfield Edinburgh Inventory. Written, informed consent was obtained prior to performing testing. This study received ethical approval from the Griffith University Human Research Ethics Committee (GU Ref No: 2020/034) and was conducted in accordance with the National Statement on Ethical Conduct in Human Research and the *Declaration of Helsinki*.

### 2.2 Experiment design

**FIGURE 1:**
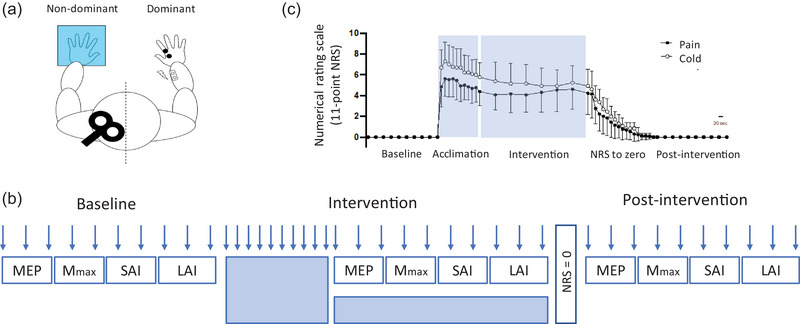
(a) First dorsal interosseous (FDI) electromyography (EMG) was measured for the dominant hand when the non‐dominant hand was immersed in water. Motor evoked potentials (MEP) were obtained from single transcranial magnetic stimulation (TMS) pulses to the primary motor cortex (M1) and Mmax was obtained from single electrical stimulations of the median nerve. Short‐latency afferent inhibition (SAI) and long‐latency afferent inhibition (LAI) were measured from paired stimulations of the motor cortex and median nerve. (b) Neurophysiological data and numerical rating scales of pain and temperature (NRS, arrows) were obtained at baseline, during the cold‐water intervention, and following the cold‐water intervention. Baseline measurements were made in 25°C water with NRS data collected every minute. An acclimation period of 5 min was employed where the same hand was immersed in 6°C cold water and NRS data were collected every 30 s. Neurophysiological measurements were then made while the hand remained in cold water. Within each block of testing, the order of MEP, SAI, LAI and Mmax data collection was randomised to avoid order effects. At the completion of the afferent inhibition measurements, participants were monitored until their NRS returned to zero for both pain and temperature. Post‐intervention measurements were then made in room temperature water with NRS data collected every minute. (c) Group NRS data are presented for temperature and pain. Data are presented as the group mean ± SD.

All participants attended a single testing session where experimental testing commenced at 10.00 h for each person. All testing was performed in a temperature controlled room where the temperature remained constant for all testing sessions. Participants were seated upright in a chair with both arms comfortably supported by an arm rest. TMS measurements were used to determine if immersion of a single limb in cold water influenced afferent inhibition of the motor cortex ipsilateral to the immersed limb (Figure 1a). First dorsal interosseous (FDI) for the non‐immersed hand was the test muscle for all measurements.

### 2.3 Timeline

At the commence of the testing session, baseline measurements were obtained when a hand was placed in 25°C room temperature water (Figure 1b). Following this, the same hand was immersed in a recirculating cold‐water bath set to 6°C. A 5‐min acclimation phase occurred where participants were encouraged to relax during the cold‐water immersion. TMS measurements were then made during the cold‐water immersion, where the participant spent no longer than 20 min in cold‐water exposure. Self‐perceptions of pain and temperature were assessed regularly throughout the testing protocol. Once the hand was removed from the cold‐water bath and placed back in room temperature water, the participants were monitored until pain and temperature returned to baseline levels. Recovery measurements were then obtained with the hand in room temperature water.

### 2.4 Electromyography

Surface electromyography (EMG) was recorded from FDI of the dominant hand using Ag–AgCl bipolar surface electrodes (24 mm, Kendall H124SG, Medtronic, Mansfield, Massachusetts, USA). The electrodes were positioned in a belly–tendon arrangement, with the ground electrode being positioned over the right ulnar styloid process and remaining in place for the duration of the experiment. Signals were amplified 300× and filtered between 20 and 1000 Hz (CED 1902, Cambridge Electronic Design Ltd, Cambridge, UK) and sampled at 2000 Hz using a 1401 data acquisition interface and Signal software (version 6, Cambridge Electronic Design). All measurements in this study were obtained when the FDI was at rest.

### 2.5 Motor cortical and median nerve stimulation

Focal transcranial magnetic stimulation (TMS) was applied to the primary motor cortex (M1) ipsilateral to the limb undergoing cold water immersion. A figure‐of‐eight stimulating coil (internal wing diameter 70 mm) was used in combination with two Magstim 200 magnetic stimulators connected with a BiStim unit (Magstim, Whitland, UK). A posterior‐to‐anterior current flow was trans‐synaptically induced by orienting the stimulating coil tangentially to the surface of the head, with the handle pointing in the posterior direction 45° to the inter‐ hemispheric line. The optimal coil location was identified as the M1 position that elicited the largest peak‐to‐peak MEPs in the test limb FDI EMG when using a slightly suprathreshold pulse. Electrical stimuli with a 100 µs pulse width were delivered to the median nerve at the wrist using a constant current stimulator (DS7AH, Digitimer Ltd, Welwyn Garden City, UK). After identifying the median nerve location with a stimulating pen, surface Ag–AgCl stimulating electrodes were placed 3 cm apart over the nerve with the distal anode electrode positioned ∼2 cm from the wrist joint.

### 2.6 Motor evoked potentials and Mmax

Single unconditioned TMS pulses were used to obtain MEPs, which were used as an indicator of corticospinal excitability. TMS intensity for experimental testing was then set to generate a MEP in the FDI EMG of ∼1 mV peak‐to‐peak amplitude during baseline conditions. Single supramaximal electrical pulses of the median nerve were used to obtain Mmax of the FDI. To achieve this, stimulator intensity was set at an intensity that was 50% above the intensity that was required to elicit a maximal compound muscle action potential in the FDI muscle at rest. Once established, the TMS and electrical stimulator intensities remained fixed throughout the duration of the experiment. Fifteen MEPs and 15 Mmax were recorded for each condition.

### 2.7 Short‐latency and long‐latency afferent inhibition

Afferent inhibition was assessed by pairing a peripheral nerve conditioning stimulus with a motor cortical test stimulus. For SAI, the conditioning electrical stimulus was delivered to the median nerve prior to a test stimulus being delivered to the motor cortex at ISIs of 18, 20, 22, 24 and 26 ms during baseline conditions. After establishing which ISI elicited the greatest inhibition for the participant, this ISI was used for the remainder of their experimental session. For LAI, the conditioning electrical stimulus was delivered to the median nerve 200 ms prior to delivering a test stimulus to the motor cortex. For all afferent inhibition trials, the conditioning electrical stimuli were set to an intensity sufficient to elicit a visible muscle contraction in FDI at baseline (∼0.2 mV), whereas TMS intensity was set to generate a MEP in the FDI of ∼1 mV peak‐to‐peak amplitude at baseline. SAI and LAI were calculated from MEP data obtained within each block of testing. Fifteen SAI and 15 LAI measures were recorded for each condition.

### 2.8 Numerical rating scales of pain and temperature

Perceived pain and cold temperature intensity were rated on a modified 11‐point numerical rating scale (NRS), with 0 indicating ‘no pain’ and ‘not cold at all’, and 10 indicating ‘worst pain imaginable’ and ‘coldest imaginable temperature sensation’. Participants also described the location, quality and intensity, of perceived pain using the McGill short form pain questionnaire.

### 2.9 Data analysis

All EMG data were analysed using functions available in Signal (version 6, Cambridge Electronic Design). MEP and Mmax amplitude were calculated from the peak‐to‐peak fluctuation in FDI EMG following a TMS pulse or electrical pulse, respectively. As there was no expectation that muscle fibres, or the peripheral nerve itself, would be affected in the non‐immersed limb, these data were not normalised and are reported as absolute values. SAI and LAI used the conditioned/test MEP amplitude ratio as the variable of interest, where a value less than 1.0 indicated that afferent inhibition had occurred (inhibition values closer to 0 indicated that the MEPs were more inhibited compared to values closer to 1.0).

### 2.10 Statistical analysis

Mauchly's test of sphericity were applied to MEP, Mmax, SAI and LAI data. Degrees of freedom were corrected using Greenhouse–Geisser corrections when sphericity was violated. One‐way repeated measures ANOVA was used to determine if the testing condition (baseline, cold‐water immersion, recovery) affected each neurophysiological variable. If a main effect of testing condition was detected, Tukey's HSD test was used to identify which condition contributed to the main effect. NRS was examined with a two‐way repeated measures ANOVA where main effects of condition (baseline, acclimation, cold‐water immersion and recovery) and scale (pain, temperature) were examined. In the event of a main effect of condition, Tukey's HSD test was used to identify which condition differed from baseline. All statistical tests were performed using SPSS Statistics (version 29, IBM Corp., Armonk, NY, YSA). Significance was set at *P* < 0.05. ANOVA effect sizes are reported as partial η^2^. Results are presented in the text and figures as means ± SD unless otherwise stated. Boxplots reflect the median and interquartile ranges, where the whiskers are 1.5× the interquartile range.

## 3 RESULTS

### 3.1 Participant adherence

Two participants did not exhibit afferent inhibition during baseline measures and were removed from the analysis. All of the remaining 13 participants were able to tolerate the intervention and testing procedures so that all neurophysiological measurements could be made.

### 3.2 Pain and temperature intensity

Participants reported no change in pain or temperature during the baseline and recovery from cold‐water immersion in the experiment. However, NRS for pain was significantly greater than baseline during the acclimation phase (*P* < 0.001) and the cold‐water immersion testing phase (*P* < 0.001, Figure 1c). Similarly, NRS for temperature was significantly greater than baseline during the acclimation phase (*P* < 0.001) and the cold‐water immersion testing phase (*P* < 0.001). The McGill pain questionnaire indicated that the most recurrent words to describe the pain associated with cold‐water immersion were sharp (92%), burning (92%), aching (77%), throbbing (77%) and heavy (62%).

### 3.3 Motor evoked potentials and Mmax

**FIGURE 2:**
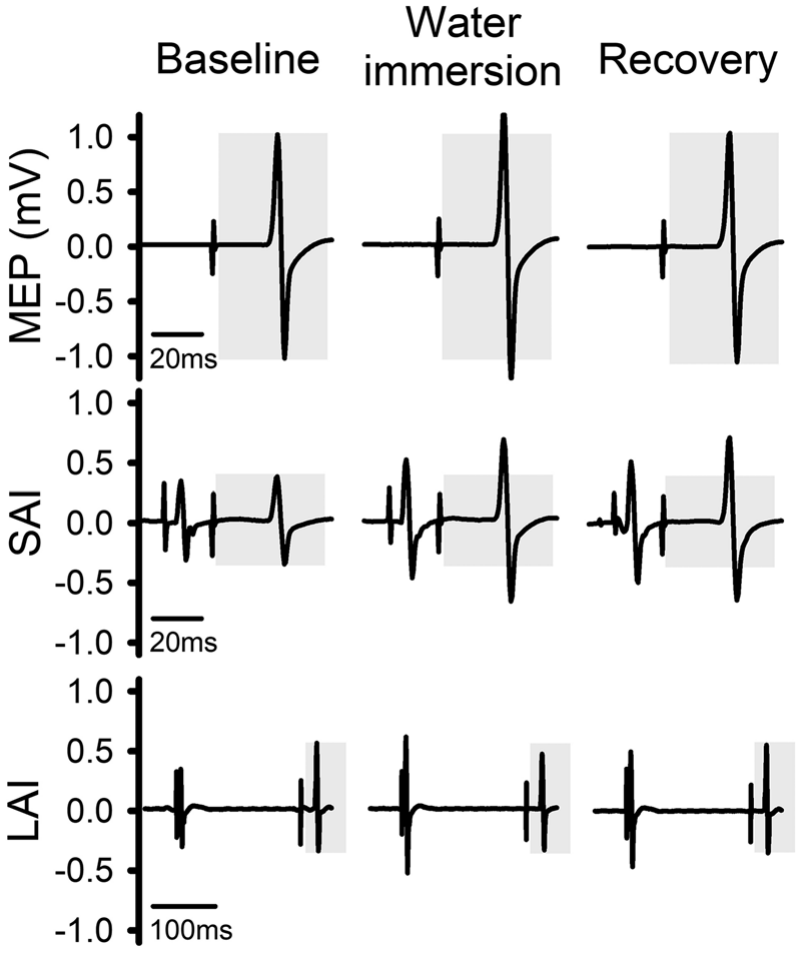
Representative EMG data for MEP, SAI and LAI of a single participant. Each variable is presented for baseline conditions, during the immersion of a single limb in cold water, and during recovery from cold water immersion after pain and temperature scales had returned to baseline values. Shaded boxes indicate the amplitude of each variable for the baseline condition.

**FIGURE 3:**
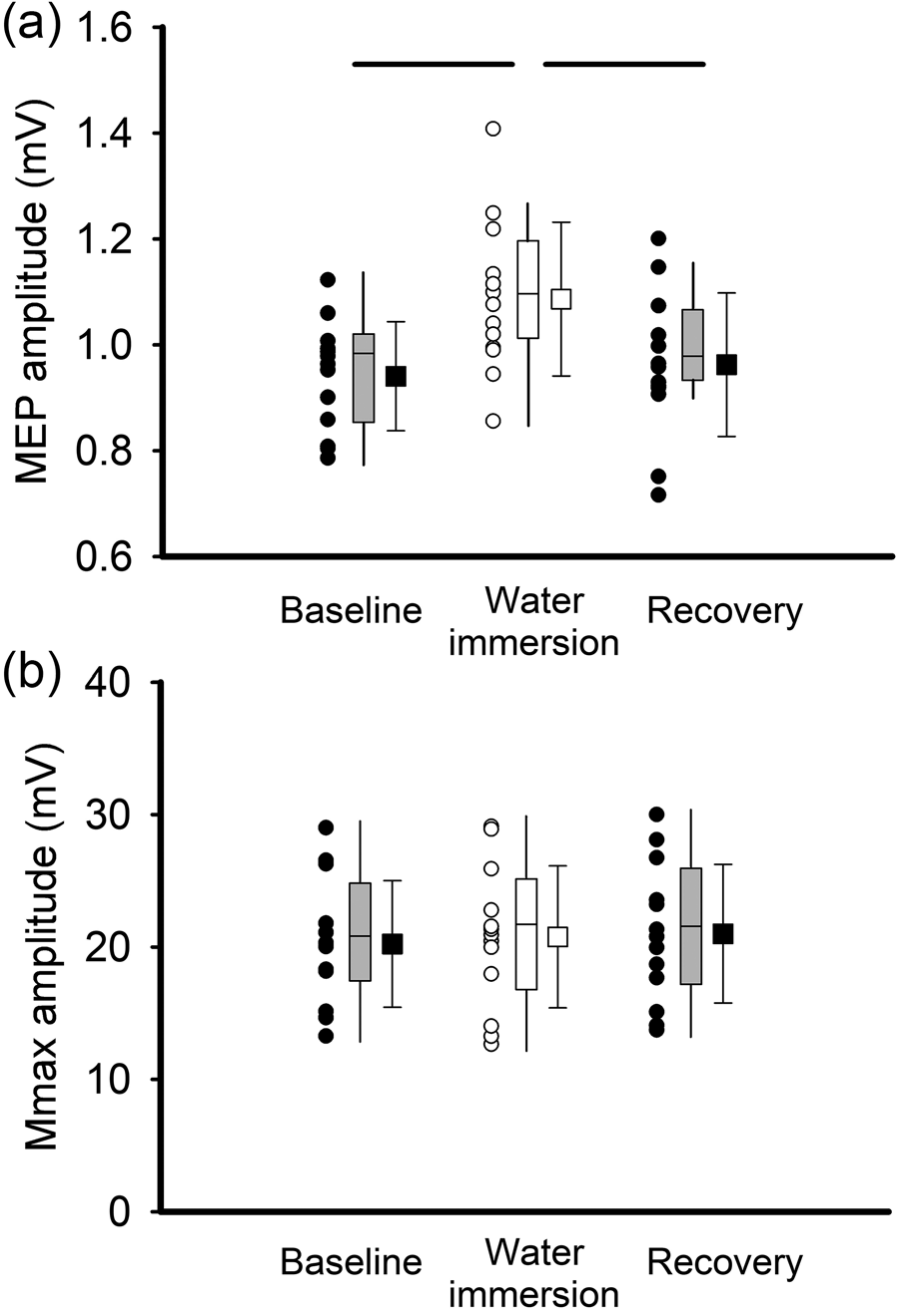
(a) MEP amplitude increased during cold‐water immersion (CWI) and then declined to approximate baseline levels following the cold‐water immersion intervention (recovery). Circles indicate individual data for each condition and squares represent the group mean (*n* = 13). Error bars represent the standard deviation of the mean, boxplots reflect the group median and interquartile range, and significance was set at *P* < 0.05. (b) Supramaximal median nerve stimulation was used to obtain Mmax in the non‐immersed limb. Mmax did not differ from baseline to cold water immersion to post‐cold‐water immersion.

Representative EMG data for the stimulation measurements obtained in this study are presented in Figure 2. A main effect of condition was detected for MEP amplitude (*F*_(1.3,15.5)_ = 8.510, *P* = 0.007, effect size (ES) = 0.415, Figure 3a). Post‐hoc analysis revealed that MEP amplitude was significantly greater during the cold‐water immersion condition compared to baseline (*P* = 0.008) and compared to recovery (*P* < 0.001). In contrast, there was no main effect of condition detected for Mmax (*F*_(2,24)_ = 2.157, *P* = 0.138, ES = 0.152, Figure 3b).

### 3.4 Short‐latency and long‐latency afferent inhibition

**FIGURE 4:**
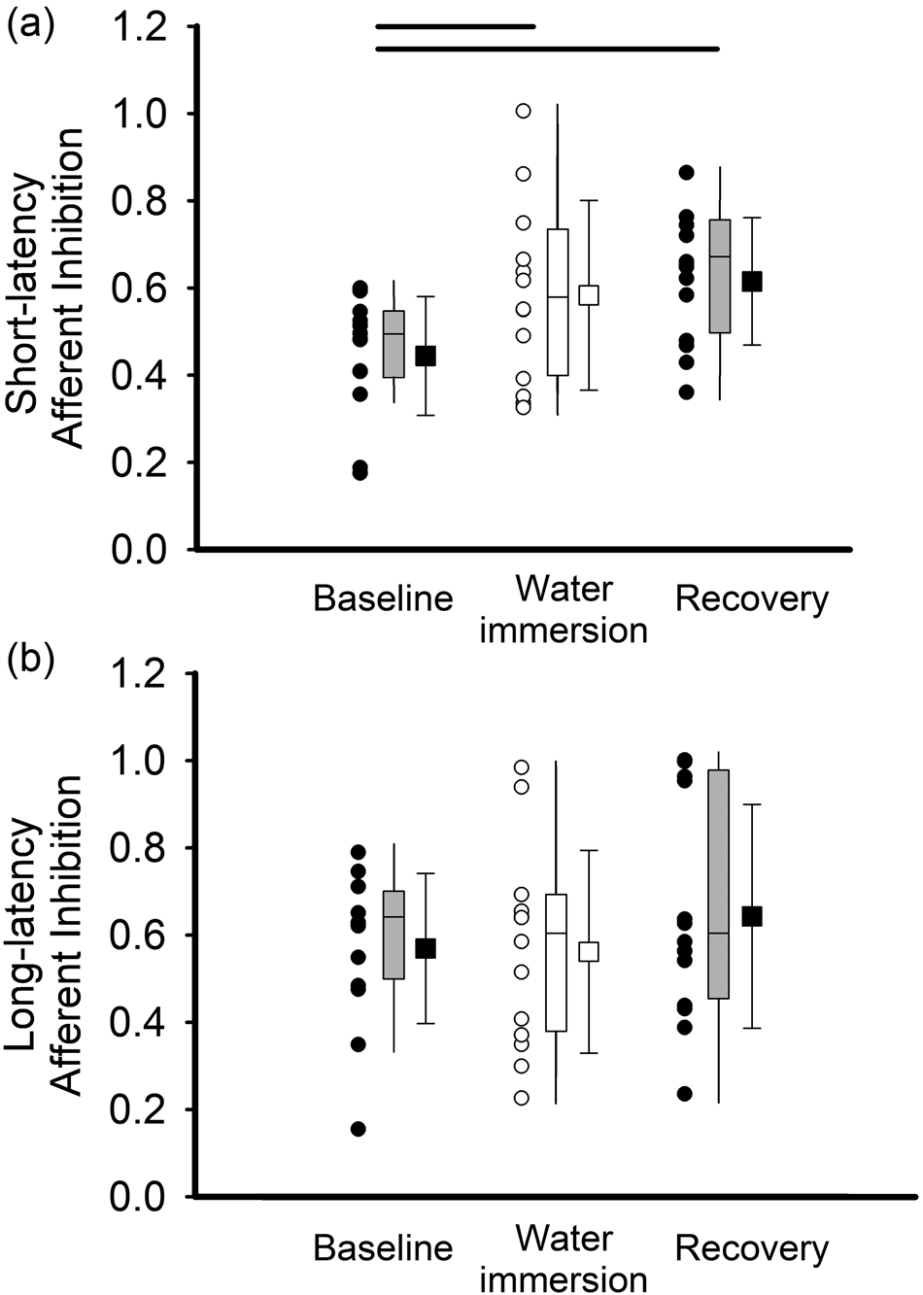
(a) SAI was measured from the FDI of the non‐immersed limb, where ISI was set to each participant's ISI that generated the greatest inhibition at baseline. SAI increased during cold‐water immersion (CWI) and then remained at similar levels of inhibition following the cold‐water immersion intervention (recovery). Circles indicate individual data for each condition and squares represent the group mean (*n* = 13). Error bars represent the standard deviation of the mean, boxplots reflect the group median and interquartile range, and significance was set at *P* < 0.05. A value of 1.0 indicated that no inhibition occurred and a value of zero indicate that the MEP was entirely inhibited. (b) LAI was also obtained in the non‐immersed limb where the ISI was set to 200 ms. LAI did not differ from baseline to cold water immersion to post‐cold‐water immersion.

The SAI ISI for each participant was set to the ISI that evoked the greatest inhibition during baseline (1 participant at 20 ms, 5 participants at 22 ms, 4 participants at 24 ms, 3 participants at 26 ms). A main effect of condition was detected for SAI (*F*_(2,24)_ = 7.647, *P* = 0.003, ES = 0.389, Figure 4a). Post‐hoc analysis revealed that inhibition was significantly reduced during the cold‐water immersion condition compared to baseline (*P* = 0.005). Similarly, inhibition was significantly reduced during recovery compared to baseline (*P* = 0.002). Unlike SAI, there was no effect of condition detected for LAI (*F*_(2,24)_ = 0.929, *P* = 0.409, ES = 0.072, Figure 4b).

## 4 DISCUSSION

The purpose of this study was to determine how immersion of a single limb in very cold water affects TMS‐evoked MEPs associated with the ipsilateral hemisphere. The strength of afferent inhibition on MEPs from the ipsilateral hemisphere was then determined via SAI and LAI measurements. Overall, there was an increase in excitability of the ipsilateral motor cortex to the limb that was immersed in water. Although there was a parallel reduction in SAI, this reduced afferent inhibition of ipsilateral hemisphere motor circuits remained during recovery when the hand was removed from the cold water.

### 4.1 MEP increases with very cold water immersion and returns to baseline in recovery

Perceptions of pain and temperature similarly increased during 6°C cold water immersion and had a modulatory effect on cortical excitability. In general, the MEP reflects central mechanisms, and Mmax reflects peripheral nerve contribution to muscle fibre activation. Thus, the dissociation between cold water immersion changes in MEP and the stability of Mmax across conditions suggests that thermally induced pain predominantly affected central rather than peripheral mechanisms of muscle activation. Although nociceptive input primarily influences the contralateral hemisphere, the ipsilateral hemisphere is also affected due to interhemispheric communication. In the current study, enhancing the sensory input to the motor cortex affected the ipsilateral motor cortex, as MEP significantly increased during cold water immersion. The increase in MEP amplitude can be attributed to the enhanced excitatory synaptic activity within the motor cortex, possibly mediated by increased glutamatergic transmission as a response to cold‐induced nociceptive and non‐nociceptive sensory input (Okano et al., 1995). The subsequent decrease in MEP amplitude upon the removal of the limb from cold water highlights a reversible adaptation of the motor cortex to the sensory inputs, potentially mediated by homeostatic plasticity mechanisms (Turrigiano, 2012). Although the effect that water immersion has on motor cortical circuitry has previously been investigated, and MEPs commonly reduce with cold‐water immersion (Burns et al., 2016a; Chowdhury et al., 2022), the current study is unique as it assessed a single limb instead of body immersion and used colder stimulus compared to other investigations (6°C compared to ∼ 20°C) (Sato et al., 2013; Yankouskaya et al., 2023).

### 4.2 SAI is reduced with water immersion and remains reduced during recovery

The stability of LAI throughout the experiment suggests that long‐latency pathways, possibly involving GABAergic neurotransmission, are less susceptible to modulation by acute sensory stimuli such as cold‐water immersion. However, changes in SAI occurred with immersion where inhibition was reduced with expose to the cold stimuli and then remained reduced even when the hand was removed from the cold water. This reduction in SAI suggests that cold water immersion leads to a sustained adaptation of cortical inhibitory circuits that synapse on motor pathways. This could be due to persistent changes in afferent input or lasting alterations in cortical neuromodulatory systems (Burns et al., 2016b; Di Lazzaro et al., 2005). Interestingly, SAI appeared to be uncoupled from MEP as there were different responses for each measurement during the recovery period. The uncoupling of changes in SAI from MEP amplitude during the recovery period suggests that the mechanisms underlying the modulation of corticospinal excitability by sensory input may be distinct from those affecting intracortical inhibitory circuits, possibly involving different sets of interneurons or neuromodulatory systems (Florian et al., 2008; Ridding & Ziemann, 2010).

The observed reduction in SAI during cold water immersion may be attributed to alterations in the cholinergic system's modulation of cortical excitability. SAI is thought to be mediated primarily by muscarinic acetylcholine receptors, which in turn modulate GABAergic inhibitory circuits within the cortex (Di Lazzaro et al., 2005; Turco et al., 2017). The persistent reduction in SAI, even after the cessation of the cold stimulus, suggests lasting changes in the excitatory–inhibitory balance within the cortex. This is potentially due to altered neurotransmitter receptor sensitivity or availability (Ziemann et al., 1996). The sustained reduction in SAI during recovery could also be linked to changes in peripheral sensory nerve function or alterations in the processing of sensory information at the spinal cord level, which in turn could affect cortical inhibitory circuits (McKay et al., 2002). This highlights the complex interaction that occurs between peripheral and central mechanisms in modulating cortical function in response to extreme temperatures that induce pain.

### 4.3 Neurophysiological basis of hemispheric interactions

Interhemispheric inhibition (IHI) is a crucial neurophysiological mechanism regulating interactions between the primary motor cortices of both hemispheres. IHI, mediated by transcallosal pathways, allows one hemisphere to exert inhibitory control over the opposite hemisphere, thus preventing unwanted movements and facilitating coordinated unilateral actions. Pain has been shown to modulate IHI significantly. Experimental pain models using hypertonic saline injections have demonstrated reduced IHI from the affected to the unaffected M1, suggesting that pain disrupts the normal inhibitory balance between hemispheres (Alhassani et al., 2019). This reduction in IHI has been observed both immediately after pain induction and at follow‐up intervals, indicating a sustained effect of pain on interhemispheric interactions (Schabrun et al., 2016). In chronic pain conditions, like lateral epicondylalgia, alterations in IHI are not consistently observed (Alhassani et al., 2024). For example, studies have shown that individuals with higher pain severity and greater disability exhibit more significant changes in IHI and sensorimotor function (Coombes et al., 2012). Collectively, these findings suggest a complex interplay between pain, cortical excitability and interhemispheric interactions, which may vary depending on the chronicity and severity of pain.

### 4.4 Clinical implications

The 6°C cold stimulus in the current evoked substantial feelings of pain, which may overlap with clinical effects of unilateral pain. Acute pain inhibition (known as endogenous analgesia or pain modulation) is an important protective mechanism that allows the individual to respond physically to a threatening or dangerous situation. In individuals with chronic or neuropathic pain, endogenous analgesia is reduced or absent. There is conflicting evidence of the effects of chronic pain on the motor cortex, but a viewpoint is emerging that increased motor cortex LICI exists in individuals with chronic pain conditions (Chang et al., 2018). Although we did not test for endogenous analgesia in response to the acute pain stimulus in our participants, it is assumed they all had an intact modulatory response, given they were young, healthy individuals. Thus, the lack of cold‐induced responses for LICI in the present study may have been a factor of healthy individuals exhibiting normal modulatory responses to acute pain.

### 4.5 Limitations

The small sample size constrains the ability to correlate MEP and SAI changes with subjective pain and temperature experiences, limiting our ability to assess the separate relationships of pain and temperature to changes in cortical excitability and afferent inhibition. In addition, the examination of contralateral hemisphere responses to unilateral sensory stimuli was not conducted due to methodological challenges in applying peripheral nerve stimulation to a submerged limb. It is important to note that our experimental design and methods were formulated to minimise the duration that participants were exposed to the extremely cold temperatures. Hence, we felt that we were not able to perform adjustments to stimulator intensity, or obtained additional variables such as resting motor thresholds, throughout the experimental conditions as this would take considerable time while participants were in pain. Importantly, there were different SAI and LAI responses to cold water immersion and recovery, which suggests that neurophysiological changes occurred due to the intervention instead of the fixed stimulator intensity for each measurement.

## AUTHOR CONTRIBUTIONS

Eden Delahunty, Leanne Bisset, and Justin Kavanagh contributed to the conception and design of the work, drafting of the manuscript, critically important intellectual content, and interpretation of data. Eden Delahunty acquired the data and performed analyses. All authors approve the results of the study and agree to be accountable for all aspects of the work in ensuring that questions related to the accuracy or integrity of any part of the work are appropriately investigated and resolved. All persons designated as authors qualify for authorship, and all those who qualify for authorship are listed.

## CONFLICT OF INTEREST

None declared.

## FUNDING INFORMATION

None.

## Data Availability

Data presented in this manuscript is available by contacting the corresponding author.
